# Can kinomics and proteomics bridge the gap between pediatric cancers and newly designed kinase inhibitors?

**DOI:** 10.1007/s00018-015-2019-7

**Published:** 2015-08-31

**Authors:** Naomi E. van der Sligte, Kim R. Kampen, Eveline S. J. M. de Bont

**Affiliations:** Division of Pediatric Oncology/Hematology, Department of Pediatrics, Beatrix Children’s Hospital, University Medical Center Groningen, University of Groningen, PO Box 30.001, 9700 Groningen, The Netherlands

**Keywords:** Targeted therapy, Kinomics, Proteomics, Drug screen approaches, Study strategies

## Abstract

The introduction of kinase inhibitors in cancer medicine has transformed chronic myeloid leukemia from a fatal disease into a leukemia subtype with a favorable prognosis by interfering with the constitutively active kinase BCR-ABL. This success story has resulted in the development of multiple kinase inhibitors. We are currently facing significant limitations in implementing these kinase inhibitors into the clinic for the treatment of pediatric malignancies. As many hallmarks of cancer are known to be regulated by intracellular protein signaling networks, we suggest focusing on these networks to improve the implementation of kinase inhibitors. This viewpoint will provide a short overview of currently used strategies for the implementation of kinase inhibitors as well as reasons why kinase inhibitors have unfortunately not yet been widely used for the treatment of pediatric cancers. We argue that by using a future personalized medicine strategy combining kinomics, proteomics, and drug screen approaches, the gap between pediatric cancers and the use of kinase inhibitors may be bridged.

## Introduction

Cancer is the second most common cause of death among children living in developed countries [1]. The current incidence of childhood cancers is 189.5 per million children and this incidence is increasing with approximately 0.6 % each year [1]. Although the 5-year overall survival rates range around 80 %, every year nearly 2000 children die due to cancer in the United States of America [1].

The introduction of chemotherapy for childhood leukemia in the beginning of the 1950s was a remarkable improvement for cancer research [2]. However, it took until 1963 and early 1970 before the first patients with acute childhood leukemia and advanced Hodgkin’s lymphoma were cured using a combination of chemotherapeutics [2]. The observed major improvements in outcome obtained over the past few decades, achieved by dose optimization and combination chemotherapy, are nowadays stagnated due to chemotherapy-related toxicity [3]. With the introduction of tyrosine kinase inhibitors (TKIs), like imatinib (Gleevec^®^), a new era of cancer therapy emerged. Imatinib transformed chronic myeloid leukemia (CML) from a fatal disease to a leukemic subtype with a favorable prognosis [4]. During the last decade, a rapid increase in the development of small molecule inhibitors and monoclonal antibodies enabled the availability for therapeutic intervention. In 2014, the US Food and Drug administration (FDA) approved 41 new drugs, of which two were protein kinase inhibitors for cancer indications (e.g., iselalisib and ceritinib, Table 1). Today, 29 protein kinase inhibitors are FDA-approved for the treatment of cancer (Table 1). Remarkably, the main targets of these approved protein kinase inhibitors are limited to the BCR-ABL kinase (six inhibitors), members of the ErbB-family receptor tyrosine kinases, especially EGFR (five inhibitors), the ALK kinase (two inhibitors), and the BRAF kinase (four inhibitors), all frequently mutated in types of adult-onset cancer [4–7].

**Table 1:** FDA-approved protein kinase inhibitors for the indication of cancer until April 1, 2015

Generic name	Trade	Target	FDA approval date	FDA-approved indications	FDA approval date for children	FDA-approved indications for children
Afatinib	Gilotrif (Boehringer Ingelheim)	EGFR, HER2, HER4	July 2013	Metastatic NSCLC (EGFR+)	NA	NA
Axitinib	Inlyta (Pfizer)	VEGFR1/2/3, PDGFR, c-KIT	January 2012	Advanced RCC	NA	NA
Bosutinib	Bosulif (Pfizer)	Bcr-Abl, Src, Lyn, Hck	September 2012	Resistant CML (Ph+)	NA	NA
Cabozantinib	Cometriq (Exelixis)	FLT3, c-KIT, c-MET, RET, VEGFR1/2/3, TrkB, Axl, Tie2	November 2012	Progressive metastatic MTC	NA	NA
Crizotinib	Xalkori (Pfizer)	ALK, c-MET, ROS1	August 2011	Metastatic NSCLC (ALK+)	NA	NA
Ceritinib	Zykadia (Novartis)	ALK, IGF-1R, InsR, ROS1	April 2014	Metastatic crizotinib-resistant NSCLC (ALK+)	NA	NA
Dabrafenib	Tafinlar (GlaxoSmithKline)	BRAF	May 2013	Unresectable or metastatic melanoma (BRAF V600E+)	NA	NA
Dasatinib	Sprycel (Bristol-Myers Squibb)	Bcr-Abl, Src, Lck, Yes, Fyn, c-KIT, EphA2, PDGFRb	June 2006	Imatinib resistant CML (Ph+) Imatinib resistant ALL (Ph+)	NA	NA
Erlotinib	Tarceva (Genentech and OSI Pharmaceuticals)	EGFR	November 2004	Metastatic NSCLC (EGFR+) Unresectable or metastatic pancreatic cancer	NA	NA
Everolimus	Afinitor (Novartis)	mTOR, FKBP12	March 2009	Unresectable SGCA (associated with TS) PNET Advanced BC (HR+, HER2−) Advanced RCC	October 2010	Unresectable SGCA (associated with TS)
Gefitinib	Iressa (AstraZeneca)	EGFR	May 2003	Advanced or metastatic NSCLC	NA	NA
Ibrutinib	Imbruvica (Pharmacyclics and J&J)	BTK	November 2013	CLL MCL	NA	NA
Idelalisib	Zydelig (Gilead Sciences)	PI3K	July 2014	Relapsed CLL Relapsed B cell FL Relapsed small lymphocytic lymphoma	NA	NA
Imatinib mesylate	Gleevec (Novartis)	Bcr-Abl, c-KIT, PDGFR	May 2001	CML (Ph+) ALL (Ph+) Unresectable or metastatic GIST	September 2006	CML (Ph+)
Lapatinib	Tykerb (GlaxoSmithKline)	EGFR, HER2	March 2007	Advanced or metastatic BC (HER2+)	NA	NA
Lenvatinib	Lenvima (Eisai)	VEGFR2/3	February 2015	Recurrent, metastatic, progressive, radioactive iodine-refractory, differentiated TC	NA	NA
Nilotinib	Tasigna (Novartis)	Bcr-Abl, PDGFR	October 2007	CML (Ph+) Imatinib resistant CML-AP and CML-BC (Ph+)	NA	NA
Palbociclib	Ibrance (Pfizer)	CDK4, CDK6	February 2015	Advanced BC (ER+, HER2−)	NA	NA
Pazopanib	Votrient (GlaxoSmithKline)	VEGFR1/2/3, PDGFR, c-KIT, FGFR1/3, Lck, Fms, Ltk	October 2009	Advanced STS Advanced RCC	NA	NA
Ponatinib	Iclusing (Ariad)	Bcr-Abl, Bcr-Abl T3151, FGFR, FLT3, VEGFR, PDGFR, Eph, Src, c-KIT, RET, Tie2	December 2012	Resistant or T3151+ CML (Ph+) Resistant or T3151+ ALL (Ph+)	NA	NA
Regorafenib	Stivarga (Bayer)	VEGFR1/2/3, Bcr-Abl, BRAF, c-KIT, PDGFR, RET, FGFR1/2, Tie2, Eph2A	September 2012	Advanced, unresectable, or metastatic GIST Metastatic CRC	NA	NA
Ruxolitinib	Jakafi (Novartis)	JAK1, JAK2	November 2011	High-risk myelofibrosis	NA	NA
Sorafenib	Nexavar (Bayer)	BRAF, c-RAF, c-KIT, FLT3, RET, VEGFR1/2/3, PDGFR	December 2005	Advanced RCC Unresectable HCC Recurrent, metastatic or progressive DTC	NA	NA
Sunitinib	Sutent (Pfizer)	PDGFR, VEGFR1/2/3, c-KIT, FLT3, CSF-1R, RET	January 2006	Progressive PNET Advanced RCC Progressed or imatinib resistant GIST	NA	NA
Temsirolimus	Torisel (Wyeth Pharmaceuticals)	mTOR, FKBP12	May 2007	Advanced RCC	May 2012	Advanced or recurrent solid cancers
Trametinib	Mekinist (GlaxoSmithKline)	MEK1/2	May 2013	Unresectable or metastatic melanoma (BRAF V600E+ or V600K+)	NA	NA
Vandetanib	Caprelsa (AstraZeneca)	EGFR, RET, VEGFR2, Brk, Tie2, EphR, Src	April 2011	Symptomatic and progressive MTC	NA	NA
Vemurafenib	Zelboraf (Roche and Plexxikon)	A/B/C-RAF, BRAF	August 2011	Unresectable or metastatic melanoma (BRAF V600E+)	NA	NA
Vismodegib	Erivedge (Genentech Inc)	Smo	January 2012	Advanced or metastatic BCC	NA	NA

Data from http://www.fda.gov/Drugs/default.htm and http://www.fda.gov/ScienceResearch/SpecialTopics/PediatricTherapeuticsResearch/default.htm

*NA* not applicable, *EGFR* endothelial growth factor receptor, *HER* human epidermal growth factor receptor, *NSCLC* non-small-cell lung cancer, *VEGFR* vascular endothelial growth factor receptor, *PDGFR* platelet derived growth factor receptor, *RCC* renal cell carcinoma, *CML* chronic myeloid leukemia, *Ph* Philadelphia chromosome, *FLT* FMS-like tyrosine kinase, *MTC* medullary thyroid cancer, *ALK* anaplastic lymphoma kinase, *ROS1* c-Ros oncogene 1, *IGF*-*1R* insulin-like growth factor 1 receptor, *InsR* insulin receptor, *EphA2* ephrin type-A receptor 2, SGCA subependymal giant cell astrocytoma, *TS* tuberous sclerosis, *PNET* pancreatic neuroendocrine tumors, *BC* breast cancer, *HR* hormone receptor, *HER2* human epidermal growth factor receptor 2, *BTK* Bruton’s tyrosine kinase, *CLL* chronic lymphoid leukemia, *MCL* mantle cell lymphoma, *PI3K* phosphoinositide 3-kinase, *B* *cell FL* follicular B cell non-Hodgkin lymphoma, *SLL* small lymphocytic lymphoma, *ALL* acute lymphoblastic leukemia, *GIST* gastrointestinal stromal tumors, *TC* thyroid cancer, *CML*-*AP* accelerated phase CML, *CML*-*BC* blast crisis CML, *CDK* cyclin dependent kinase, *ER* estrogen receptor, *STS* soft tissue sarcoma, *FGFR* fibroblast growth factor receptor, *CRC* colorectal cancer, *HCC* hepatocellular carcinoma, *DTC* differentiated thyroid carcinoma, *CSF*-*1R* colony stimulating factor 1 receptor, *EphR* ephrin receptor, *Smo* smoothened, *BCC* basal cell carcinoma

Protein kinase inhibitors suppress the activity of kinases, enzymes catalyzing protein phosphorylation by transferring phosphate groups from adenosine triphosphate (ATP) to specific proteins. Protein kinases are attractive targets for cancer therapy, as the malignant transformation of cells highly depends on deregulated kinase-mediated signal transduction pathways; intracellular signaling cascades involving protein phosphorylation events regulating critical cellular processes [8, 9].

Focusing on FDA-approved protein kinase inhibitors for children revealed an approval of only three inhibitors (Table 1). To date, several drugs that have been approved for the treatment of adult malignancies are often only prescribed off-label for the treatment of pediatric cancer patients. However, the extrapolation of clinical trial results obtained from treating adult patients towards pediatric cancer patients is often inappropriate [10]. First, malignancies in children are different compared to adult malignancies [10]. Secondly, medications metabolize differently in children compared to adults, resulting in unpredictable treatment responses and side effects in children [10]. Pediatric drug testing is problematic for a number of reasons. Clinical trials in children are restricted to diseased children for whom a minimal benefit of participating in the clinical trial should be achieved. Furthermore, in contrast to trial participation in adults, parents and pediatricians are usually more concerned about the risks and benefits for the individual child [10]. The most important reason why clinical trials in children have been hampered is the limited number of patients eligible for clinical trials, since pediatric cancer is relatively rare. Moreover, as a consequence of these low patient numbers, the pharmaceutical industry is less interested in funding clinical trials in children since pediatric clinical trials are costly and the financial profit is minimal [10]. Nonetheless, we have to prevent that ineffective and potentially harmful interventions are subjected to pediatric oncology patients before they have been properly tested.

To improve pediatric medicine, pediatric regulations came into force in the European Union in 2007 and the Pediatric Investigation Plan (PIP) was launched; a research and development program aimed at ensuring the generation of data required to determine the conditions in which a compound may be authorized to treat the pediatric population [11, 12]. As a reward for participating in the PIP, pharmaceutical companies gain patent extension. The introduction of these regulations has resulted in more pediatric clinical trials, an increase in available drugs authorized for pediatric indications, and prevented that children are subjected to unnecessary studies [11, 12]. Nevertheless, still only three protein kinase inhibitors are approved for the treatment of pediatric malignancies.

To summarize the current problem, on the one hand we have a multitude of small molecule inhibitors including protein kinase inhibitors (either FDA approved or still in the pipelines of pharmaceutical companies), and on the other hand we have a number of children with untreatable cancer. Since we face limitations implementing these kinase inhibitors for the treatment of pediatric malignancies, many potentially useful drugs remain unused. This viewpoint will provide (1) a short overview of study strategies, including genome and transcriptome profiling, kinome and proteome profiling, and drug screen approaches currently used to gain insight into intracellular signaling networks that are of potential interest for the introduction of new treatment options, and (2) highlights the reasons why kinase inhibitors are unfortunately not commonly used for the treatment of pediatric cancer. Lastly, we will propose a personalized medicine strategy by combining kinomics, proteomics, and drug screens aiming to bridge the gap between pediatric cancers and the use of kinase inhibitors.

## Hallmarks of cancer

Cancer can arise in different organs, tissues, and cell types, all with a distinct disease presentation and outcome. Several characteristics are shared throughout different cancers. These characteristics, or key hallmarks of cancer, were established by Hanahan and Weinberg presenting the complexity and capabilities of cancer cells [13, 14]. The hallmarks of cancer comprise: sustaining proliferative signaling, evading growth suppressors, resisting cell death, enabling replicative immortality, inducing angiogenesis, activating invasion and metastasis, avoiding immune destruction, tumor-promoting inflammation, genome instability and mutation, and deregulating cellular energetics. These diverse processes have in common that they provide a growth advantage of the cancer cell compared to its normal counterpart. Recent technologies such as genome-wide genetic and transcriptional analysis using next-generation sequencing revealed the mutational landscape of many adult and pediatric cancers [15–18]. These thorough analyses led to the discovery that, in general, pediatric cancers exhibit fewer mutations than adult cancers, and that within specific types of cancer there is a high variability of the mutations present [15]. This knowledge leaves us with the thought that CML, harboring one unique and uniform driver mutation (namely, *BCR*-*ABL*), is in fact an exceptional situation. In other malignancies, the pathobiology is more complex. For example, despite intensive genome and transcriptome profiling, the majority of the pediatric acute lymphoblastic leukemia (ALL) cases remain without explanation of precise genetic etiology [18]. Therefore, the question must be asked; how can we bridge the gap from insights in the hallmarks of cancer to the use of available kinase inhibitors that are on the market?

In addition to genetic alterations, epigenetic alterations and the influence of microenvironmental factors can contribute to oncogenesis and disease progression. Many of these alterations ultimately support somatic cells to escape the restraints that normally withhold them from unlimited cell proliferation. This growth advantage is the net result of aberrant activated signal transduction pathways [8]. Within cancer cells, the above-mentioned hallmark capabilities are regulated by highly interconnected intracellular signaling networks [14]. Therefore, to understand how specific kinase inhibitors may affect cancer hallmarks, more insights into the key proteins within the intracellular signaling networks to which these drugs can counteract are needed. Currently, many recent studies focus on generating insights into signal transduction networks as a final common pathway of various cancer hallmarks that are translated into cancer cell progression.

## Short overview of strategies used for the identification of treatment options

### Genome and transcriptome profiling

In the last decade, genome and transcriptome sequencing have improved our understanding of human cancers significantly [15, 19]. These cancer studies have revealed a set of 138 genes that, when altered by mutations, can promote oncogenesis [15]. Additionally, mutations can be theoretically distinguished in “driving mutations”, mutations that confer a selective growth advantage, or “passenger mutations”, mutations that have no effect on neoplastic processes [15]. In practice, it is difficult to determine which mutations drive oncogenesis and/or contribute to malignant transformation or progression [15]. This is partially due to the fact that some mutations require collaborative mutations to enable oncogenic transformation. Importantly, comprehensive sequencing efforts have revealed genetic alterations that are now being treated with specific kinase inhibitors. This started with BCR-ABL inhibitors for the treatment of CML and has continued with, for example, *ALK* translocations and *EGFR* mutations in non-small-cell lung cancer and *BRAF* mutations in melanoma (Table 1), as well as genetic alterations currently tested in clinical trials such as *FLT3*-*ITD* and *JAK* mutations in pediatric leukemias [20–22]. Unfortunately, although genome and transcriptome profiling has increased our understanding of oncogenesis and improved outcome for several malignancies, there are still malignancies, especially in children, in which the oncogenic alterations that drive cancer progression are largely unknown. Consequently, kinase inhibitors directed to specific driving mutations cannot be used for the treatment of most pediatric malignancies. Moreover, the combination of gene expression signatures and anticancer drug sensitivity patterns provided inconsistent results. While some cancers with known mutations expected to be sensitive towards specific inhibitors proved indeed to be highly sensitive, in other cancers, harboring the same mutation, specific inhibitors presented minor anticancer responses or respond only for a short period of time [23–25]. Additionally, the group of Clevers recently showed a gene-drug association of only ~1 % between individual oncogenic mutations and drug response in adult colorectal carcinoma patients [26]. Therefore, additional study strategies are required for the identification of suitable targets for therapy.

### Kinome and proteome profiling

**Fig. 1:**
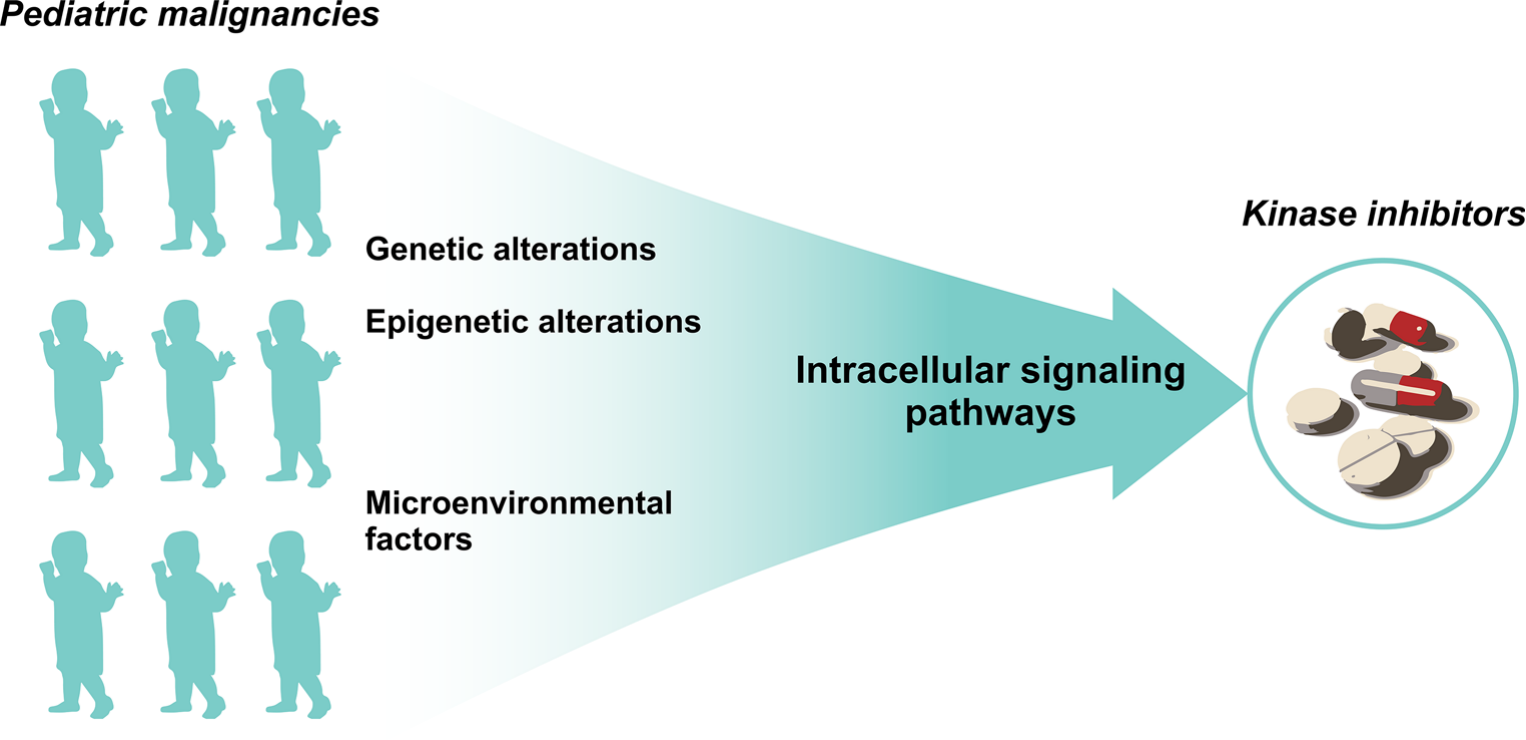
An illustration of why insight into intracellular signaling pathways might be a potent strategy for bridging the gap between pediatric malignancies and the use of available kinase inhibitors

As genetic and epigenetic alterations and/or microenvironmental factors ultimately influence the activation of intracellular signaling networks, insight into these intracellular signaling pathways might be a potent strategy for identifying targetable signaling hubs for the treatment with kinase inhibitors (Fig. 1) [8]. Current techniques to study protein phosphorylation include for example high-throughput techniques as reverse phase protein arrays (RPPA), more intensive analysis by mass-spectrometry, or single cell probe-based flow cytometry. Additionally, the activity of kinases might be studied by for example high-throughput peptide-based kinase activity arrays [27]. We recently provided an extensive overview of the pros and cons for different proteomic techniques that aim to assess protein kinase activation and protein phosphorylation [27]. Elucidating signaling networks to identify suitable targets for therapy might be valuable particularly for children since pediatric cancers harbor fewer mutations compared to adult cancers [15]. In recent years, our lab focused on using comprehensive kinome and proteome profiling to identify signaling networks as well as potential druggable targets for various pediatric malignancies [28–30]. These studies showed that kinome profiling is an elegant approach for identifying therapeutic targets by elucidating signaling pathways for common pediatric cancers, e.g., leukemias [ALL and acute myeloid leukemia (AML)] and brain tumors [28–30]. For example, kinome and proteome profiling revealed c-AMP-responsive element binding protein (CREB) activity in pediatric ALL, MEK activation in pediatric MLL-rearranged AML, Src activity in pediatric brain tumors, and a role for Eph/ephrin signaling in pediatric medulloblastoma [28, 29, 31, 32]. All these findings were validated using in vitro cytotoxicity screens that confirmed their potential as a therapeutic target [28, 29, 31, 32]. More importantly, we showed that the combination of kinome and proteome profiling is a powerful prediction approach for signaling pathway adaptations and redundant pathway discovery upon single targeted therapy and can be used to define rational combination therapies [29]. For example, this approach revealed activity of the MAPK and PI3K/Akt/mTOR signaling pathways in pediatric MLL-rearranged AML and predicted that a sustained PI3K/Akt/mTOR pathway activation enabled a subpopulation of cells to survive MEK inhibition [29].

### Drug screen approaches

Drug screening and genetic knockdown approaches, such as high-throughput RNAi and kinase inhibitor screens, have been used to define kinase pathway dependence [33–37]. These strategies created patient-specific in vitro sensitivity profiles against specific kinase targets by treating adult primary leukemia cells with siRNA or kinase inhibitors [35, 37]. Two important conclusions could be drawn based upon these results, namely (1) there is a great heterogeneity in predicted kinase targets between patients, even within similar diagnostic subgroups, and (2) the oncogenic mechanism for predicted therapeutic targets could not be elucidated based upon underlying genomic alterations [33, 35]. For example, gene silencing identified an upregulation of receptor tyrosine kinase-like orphan (RO)R1 expression in t(1;19)-positive pediatric ALL patients, a mechanism based on pre-B cell receptor signaling inhibition rather than ROR1 activating mutations or aberrant transcription profiles [38]. Most importantly, drug screens showed that targeting intracellular signaling pathways is a feasible therapeutic option.

As a proof of concept, Tyner and Pemovska used the results from their drug screen to treat adult leukemia patients not eligible to standard treatment options using FDA-approved kinase inhibitors (*n* = 1 and *n* = 8, respectively) [35, 37]. The initial results were very promising, showing rapid decreases in white blood cell counts and bone marrow blast counts in the majority of the patients. However, effects were only short lasting; within months patients relapsed after personalized kinase inhibitor treatment [35, 37]. Repeated drug screens, of the relapsed leukemia samples, showed resistance to the initially used kinase inhibitors as compared to their corresponding pretreatment samples [35, 37]. These examples illustrate why most long-term clinical results of kinase inhibitors are disappointing when using monotherapy [39]. Innate or acquired cellular resistance to kinase inhibitors are a major clinical challenge [24, 35, 37, 40].

## Resistance to kinase inhibitors

Several mechanisms of cancer cell resistance to kinase inhibitors have been described. First of all, advanced alterations in the present mutation, for example new kinase domain mutations, confer resistance to kinase inhibitors by decreasing the efficiency of the inhibitor [41]. A classic example is *BCR*-*ABL1* kinase domain mutations decreasing the sensitivity to imatinib in CML [42].

Secondly, newly acquired alterations might circumvent the inhibitory effect of a given drug; for instance, the accumulation of various new genetic abnormalities in CML result in the activation of signaling pathways independent of BCR-ABL activity and consequently facilitates disease progression to blast crisis [41, 43, 44]. Similarly, mutations in *MEK1* can confer resistance to BRAF inhibition [45].

Thirdly, therapy resistance can be mediated by cellular adaptations through dynamic reprogramming, e.g., the activation of alternated routes of kinase pathway activation in response to pharmacological inhibition [46]. Cellular adaptation by dynamic reprogramming is an important challenge for the implementation of kinase inhibitors and can occur by either the reactivation of the targeted pathway or via bypass opportunities through the activation of alternative signaling pathways [41, 46]. An example of reactivation of the targeted pathway is *B*-*RAF (V600E)*-positive melanoma [47]. These cells can acquire resistance to vemurafenib by reactivating the MAPK pathway via N-RAS upregulation. Dynamic reprogramming might also result in the activation of alternative signaling pathways; for instance, by the upregulation of RTK-ligand levels that has been frequently observed following kinase inhibition and is able to activate downstream highly interconnected intracellular signaling pathways, most notably the PI3K/Akt/mTOR or MAPK signaling networks [40, 46]. In addition, resistance to the BRAF inhibitor in colorectal carcinoma might be due to the activation of the PI3K/Akt/mTOR signaling pathway [48].

Finally, intratumor heterogeneity can also restrict the implementation of targeted therapy. Intratumor heterogeneity may lead to inferior therapeutic responses to kinase inhibitors since it has been established that the outgrowth of a therapy-resistant subclone(s) can lead to refractory or relapsed disease [41].

## Future strategy for clinical trials in pediatric oncology

Taken together, this viewpoint has highlighted currently used strategies for the implementation of kinase inhibitors as well as reasons why kinase inhibitors have unfortunately not yet been widely used in pediatric cancer therapy. In this paragraph, we will propose a personalized medicine strategy attempting to improve the implementation of kinase inhibitors in pediatric cancer.

**Fig. 2:**
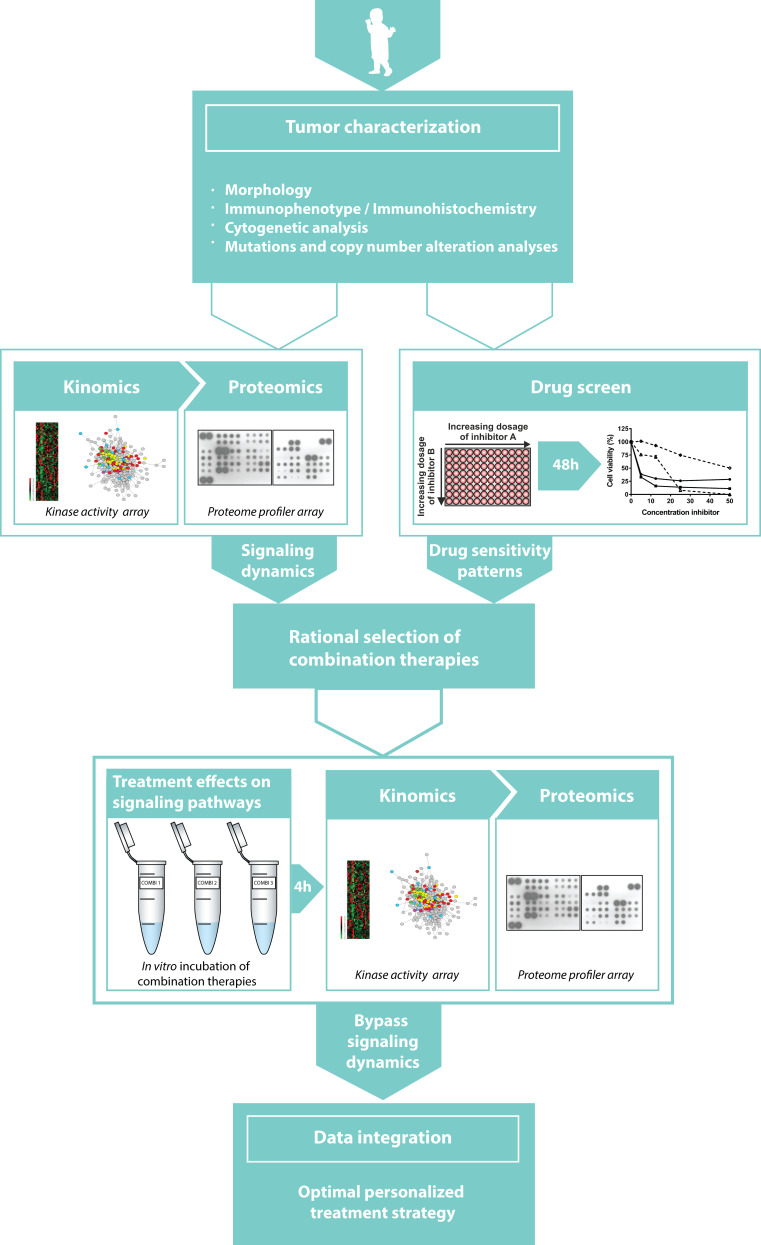
Visualization of a future personalized medicine strategy attempting to improve the implementation of kinase inhibitors in pediatric cancer. After initial tumor characterization, we propose to perform kinome and proteome profiling on patient samples, as well as subject patient cells to a drug screen including multiple kinase inhibitors (either FDA approved or in the pipelines of pharmaceutical companies) to characterize their patient-specific cancer profile. Integrating these results will define rational combination therapies. To determine treatment effects on signaling, kinome and proteome profiles will be re-determined after in vitro treatment with potential combination therapies. Ultimately, data integration of all these multilevel study elements will result in a comprehensive network of pre-treatment active signaling pathways, putative targets for targeted therapy, and subsequent post-treatment drug-induced bypass mechanisms for cellular resistance

We argue that establishing the active intracellular signaling pathway networks in cancer patient samples will be a suitable strategy in deciding which kinase inhibitors (either FDA approved or in the pipelines of pharmaceutical companies) should be used to target the cancer cell (Fig. 1). As previously mentioned, drug screens have initially showed promising short-term results towards this end [33–37]. Further, we have demonstrated that kinome and proteome profiling is an elegant approach for identifying potential druggable targets in pediatric malignancies [28–30]. Additionally, we showed that this strategy is able to predict signaling pathway adaptations that can be used to define rational combination therapies, as shown for combined MEK and VEGFR-2 inhibition in pediatric MLL-rearranged AML [29]. Combining these kinomics and proteomics study approaches with a comprehensive drug screen can define major contributing protein kinases relevant for cancer cell survival (Fig. 2). Following upon initial tumor characterization, we propose to perform kinome and proteome profiling to determine networks of active signaling pathways, which enables to extract key signaling hubs and also provides insight into how to predict possible cancer cell bypass mechanisms based upon signaling availability. Additionally, cancer cells will be subjected to a drug screen containing drugs in current use for cancer treatment, drugs previously investigated in or currently undergoing clinical trials, and experimental compounds to characterize cancer cell-specific drug sensitivity patterns. While drug screens are relatively easy to perform for hematological malignancies, the implementation of drug screens for solid tumors is more challenging—but not impossible. Recently, it has been shown that an organoid culture platform can be used for functional drug screening assays of solid cancers [26]. This model also reflects the polyclonality of tumors enabling a suitable predictive model to define cytotoxic responses to therapy at the level of the individual patient [26]. Integrating the kinome and proteome profiles together with drug sensitivity profiles into one network will generate an overview of highly active signaling pathways including the corresponding putative novel targets for therapy (highlighted in the network, Fig. 2). Based on this network, rational combination therapies could be defined by selecting suitable targets from different signaling pathways.

Since cellular dynamic reprogramming and intratumor heterogeneity are major challenges for the implementation of kinase inhibitors, insight into the adaptive kinome responses and subclonal resistance to kinase inhibitors is essential. By doing so, one can anticipate on mechanisms of resistance. As it has been shown that redundant signaling pathways as well as signaling profiles of minor subclones are not per se detectable at the time of diagnosis and might become more prominent after treatment with specific kinase inhibitors, it will be necessary to re-determine cellular dynamics of signaling pathway activation after in vitro treatment with selected combination therapies (Fig. 2). This second network analysis of signaling pathways might reveal cellular adaptations by activating signaling events that can facilitate therapeutic resistance.

Integration of all these multilevel study elements will generate a comprehensive network of pre-treatment active signaling pathways, putative targets for targeted therapy, and subsequent post-treatment drug-induced bypass mechanisms for cellular resistance. If necessary, the initial selected combination therapies can be modified to circumvent drug-induced bypass signaling pathways and to select an optimal therapeutic strategy in advance. Extrapolation of this proposed in vitro model to an in vivo model increases the translational feasibility of the preclinical treatment screening, which is highly desirable since only 5 % of the identified putative anticancer compounds present sufficient clinical activity in phase III trials [49, 50]. In the meantime, optimization of in vitro models, for example by tumor organoid cultures, is of great importance to improve preclinical models for drug testing.

One additional problem that we have to overcome is the low number of pediatric patients eligible for clinical trials. We have noticed an overlap of recurrent active signal transduction pathways within different subtypes of cancer [28–30]. Furthermore, the kinase inhibitor screen of Tyner et al. showed no complete segregation based upon leukemia subtypes [35]. Therefore, we propose that all children suffering from cancer without evidence-based treatment options are eligible to enroll in this study strategy. Combining different patient populations allows studying the mechanism of signal transduction adaptations and the rational design of combination therapies in a significant larger cohort of children. More importantly, a trial including children with comparable signaling dynamics will provide information about the optimal biological dose for the kinase inhibitor; the dose that produces a quantifiable effect in inhibiting the target in the cancer cells (primary endpoint). Additionally, pharmacokinetics, pharmacodynamics, side effects, and toxicity spectrum of the specific inhibitor in pediatric oncology patients should be included as important objectives. Moreover, while the primary objective of the proposed study design is bridging the gap between pediatric cancers and newly designed kinase inhibitors to the improve survival of children without evidence-based treatment options, consequent studies regarding the long-term effects of kinase inhibitors on energy metabolism, growth and bone mineral density, gonadal function and reproduction, and cardiac health are warranted.

Finally, since the continuous development of new study approaches is essential for the implementation of targeted therapies, we expect that the proposed pre-clinical screening strategy should incorporate additional novel methods according to new developments. This integrated multilevel screen might easily be developed further to an integrated model of genome, kinome, and proteome profiling, supported with networks of cell–cell and cell–stroma interactions. In conclusion, despite initial disappointing results of kinase inhibitors in clinical trials, we propose that available kinase inhibitors holds tremendous promise for most malignancies when using novel selective combinations of therapeutic interventions. In this viewpoint, we illustrate a personalized medicine strategy combining kinomics and proteomics approaches with a comprehensive drug screen to define rational combination therapies that may bridge the gap between pediatric cancers and the implementation of kinase inhibitors.
